# Circular RNA expression alterations and function prediction in OSA-induced pancreatic injury in mice: New insights into pathogenesis

**DOI:** 10.1371/journal.pone.0284459

**Published:** 2023-04-14

**Authors:** Qingshi Chen, Jiayu Lin, Zhiyu Chen, Lishuang Che, Dexin Liu

**Affiliations:** 1 Department of Endocrinology and Metabolism, The Second Affiliated Hospital of Fujian Medical University, Quanzhou, China; 2 Department of Radiology, The Second Affiliated Hospital of Fujian Medical University, Quanzhou, China; 3 Department of Nephrology, The Second Affiliated Hospital of Fujian Medical University, Quanzhou, China; University of Nebraska Medical Center, UNITED STATES

## Abstract

**Objectives:**

Increasing studies have shown that circular RNAs (circRNAs) participate in the pathogenesis and progression of many diseases. However, the function of circRNAs in obstructive sleep apnea (OSA)-induced pancreatic damage has not been fully elucidated. In this study, the altered circRNA profiles in a chronic intermittent hypoxia (CIH) mouse model were investigated, aiming to provide novel clues for delineating the underlying mechanisms of OSA-induced pancreatic injury.

**Methods:**

A CIH mouse model was established. circRNA microarray was then applied to profile circRNA expression in pancreatic samples from CIH groups and controls. Our preliminary findings were validated by qRT-PCR. Subsequently, GO and KEGG pathway analyses were carried out to annotate the biological functions of target genes of circRNAs. Lastly, we constructed a circRNA-miRNA-mRNA (ceRNA) network according to the predicted circRNA–miRNA and miRNA–mRNA pairs.

**Results:**

A total of 26 circRNAs were identified to be differentially expressed, with 5 downregulated and 21 upregulated in the CIH model mice. Six selected circRNAs were preliminarily used to confirm the results by qRT-PCR, which were consistent with microarray. GO and pathway analysis indicated that numerous mRNAs were involved in the MAPK signaling pathway. The ceRNA analysis displayed the broad potentials of the dysregulated circRNAs to modulate their target genes by acting as miRNAs sponges.

**Conclusions:**

Taken together, our study first revealed the specific expression profile of circRNAs in CIH-induced pancreatic injury, which suggested a novel focus for investigating the molecular mechanism of OSA-induced pancreatic damage through modulating circRNAs.

## Introduction

Obstructive sleep apnea (OSA) is a rather common chronic sleep disorder with a key character of chronic intermittent hypoxia (CIH). Long-term untreated OSA may result in multiple health impairments, such as lung damage [1], kidney injury [2], metabolic disorders [3], and cardiovascular disease [4], which compromises the quality of life and brings psychological and economic burdens to patients. Moreover, OSA is closely related to glucose metabolism disorder. It is also recommended as an independent risk factor for type 2 diabetes mellitus [5, 6]. Previous results suggested that OSA could lead to insulin secretion disorder and pancreatic injury in rats with CIH exposure [7]. Meanwhile, CIH promotes the production of large amounts of reactive oxygen species (ROS), which results in insulin resistance and glucose intolerance. However, studies about the effects of OSA on insulin, pancreatic cells, and diabetes are still very few, and the molecular mechanism remains unclear.

Circular RNAs (circRNAs) are a class of novel noncoding RNAs, characterized by stability, conservation, and tissue‑specific expression [8, 9]. Originally, researchers paid little attention to circRNAs and the relevant studies on circRNAs were still in their infancy. However, emerging evidence demonstrated that altered changes of circRNAs were linked to a variety of human diseases, including cardiovascular disease [10], neurodegenerative diseases [11], intracranial aneurysms [12], and cardiac regeneration [13]. With the development of biotechnology, we then gradually revealed the characteristics of circRNAs. Recent studies reported that circRNAs could function as a miRNA sponge and regulate targeted gene expression [14–16]. In addition, circRNAs dysregulation was engaged in numerous cancers, including colorectal cancer [17], breast cancer [18], bladder cancer [19], gastric cancer [20], and liver cancer [21]. However, few studies have investigated circRNA alterations in a CIH injured pancreas.

In the present study, circRNA microarray analysis was applied to profile expressions of circRNAs in the CIH-induced pancreatic injury mouse model. The expression levels of several differentially expressed circRNAs were further validated by qRT-PCR. Bioinformatic analyses and circRNA-miRNA-mRNA interaction networks were conducted to predict the potential role of circRNAs in the CIH-induced pancreatic damage. Together, our study first revealed the expression profiles and potential functions of altered circRNAs in a CIH-injured pancreas in mice, which may serve as novel targets for clinical therapy of OSA-induced pancreatic injury in humans.

## Materials and methods

### Animal

BALB/c mice (Weitong Lihua, Beijing, China) were used in the present study. The study was performed according to the Guide for the Care and Use of Laboratory Animals issued by the Institute of Laboratory Animal Resources of the Life Science Committee of the National Research Council. The Research Ethics Committee of the 2nd Affiliated Hospital of Fujian Medical University approved of the study protocol. All of the mice were fed with free access to water and food.

### CIH protocol

The model of CIH was established using our previously described protocol [22]. Briefly, mice assigned to the CIH group were placed in a specially designed chamber. The chamber was connected to a gas control delivery system which allows the flow of pure nitrogen to reduce O_2_ to 6% within 60 seconds, after which the control system also allows a quick replacement of oxygen resulting in a rapid reoxygenation to 21% O_2_ within 60 seconds. This 2-minute CIH cycle was repeated 30 times per hour for 8 hours per day, for a total duration of 8 weeks. For the Control group, room air was forced into the chamber during the entire experiment. After exposure to CIH for 8 weeks, all mice were euthanized by cervical dislocation by a trained professional after isoflurane anesthesia. Then, the pancreatic tissues were isolated.

### Hematoxylin-eosin (HE) staining

The pancreatic tissues harvested from the mice were washed with 0.9% saline, fixed in 4% formaldehyde, and then embedded in paraffin. Finally, the tissues were sliced to 5μm thick and stained with HE for further analysis. The morphologic alterations were observed under a light microscopy.

### CircRNA microarray analysis

We have finished the Arraystar Mouse circRNA Array V2 analysis of the 6 samples. According to the standard protocols Arraystar (Arraystar, Inc.), we conducted the work of sample labeling and microarray hybridization. In brief, we first used Rnase R (Epicentre, Inc.) to digest the total RNAs. Then, we utilized a random priming method (Arraystar Super RNA Labeling Kit) to amplify the enriched circRNAs and transcribe them into fluorescent cRNA. The labeled cRNAs were next hybridized to Mouse circRNA Array V2 (Arraystar). Subsequently, the Agilent Scanner G2505C was used by us to scan the arrays. We employed the Agilent Feature Extraction software (version 11.0.1.1) to analyze the images. circRNAs were deemed as significantly dysregulated by the cutoff fold change≥1.2 and P<0.05. At last, we applied the R software limma package to perform quantile normalization and subsequent data processing.

### GO and KEGG pathway analysis

GO and KEGG enrichment analysis were performed to reveal the potential role of circRNAs. We presented the enriched GO terms by using enrichment score. KEGG analysis was applied to determine the different biological pathways of downstream mRNAs. The threshold for the P‑value was <0.05 and the count number was >2. The enrichment score [–log10 (p-values)] represented the significance of GO and KEGG pathway correlations.

### Construction of circRNA/miRNA interaction network

We predicted the miRNA target sites on circRNA with the use of Arraystar’s homemade miRNA target prediction software, which was based on TargetScan and miRanda. According to the seed match sequences, the circRNA-miRNA network was further constructed. The network was drawn by using Cytoscape 3.6.1.

### qRT-PCR

We performed qRT-PCR to evaluate the expression level of 6 candidate key circRNAs by using one-step SYBR^®^ primescript^TM^ RT-PCR kit (TaKaRa, China) on Applied Biosystems 7500 FAST (USA). The primers were synthesized by Sangon Biotech (Shanghai, China, Table 1). The specificity of PCR primers was verified by a single-peak appearance on the melting curve. The 2^−ΔΔCT^ method was used to analyze relative circRNA expression normalized to GAPDH. All qRT-PCRs were performed in triplicate.

**Table 1:** Primers designed for qRT-PCR.

Gene name	Forward and reverse primer	Tm (°C)	Amplicon length (bp)
GAPDH	F:5’CACTGAGCAAGAGAGGCCCTAT3’	60	144
	R:5’GCAGCGAACTTTATTGATGGTATT3’		
mmu_circRNA_38000	F:5' GAGCATCTGGAGAAGGTGGTG3’	60	184
	R: 5’ TCGAATCAAAGCTGGGAGGT 3’		
mmu_circRNA_015198	F:5' AGAGCAATTCCTGAACCTCGTC 3’	60	162
	R: 5’ TCCAGGTTAAAGTGGGTCTCG3’		
mmu_circRNA_31565	F:5'GTGTAACCTGATCACAGACAAAG3’	60	66
	R: 5’ CTATCAACTGTTTCGTCATCCT3’		
mmu_circRNA_36426	F:5' TGATGGATAAAGCCAAGGGTC3’	60	212
	R: 5’ TTCTTCAAATGCGTTCACTGC 3’		
mmu_circRNA_39531	F:5' GGGGTCCTGACCTACTGGTT3’	60	121
	R: 5’ CTGTGGCACTGGTGTCTTCG 3’		
mmu_circRNA_36670	F:5' CAACCCTCAGTCCTCAGTCG3’	60	51
	R: 5’ TTCCCTCCTACAGTCTCCCAT 3’		

### Construction of a ceRNA network

Based on the ceRNA hypothesis, a circRNA-miRNA-mRNA network was constructed by using Cytoscape 3.6.1. With the known targeted miRNAs of each circRNA, we then predicted their downstream target genes based on TargetScan & miRanda. Finally, the ceRNA network was created by the use of the validated six circRNAs and their predicted miRNAs/mRNAs.

### Statistical analysis

For comparisons of circRNAs expression levels between the CIH and control group, all data were calculated using the SPSS 20.0 with a Student’s t-test. All experiments were conducted three times. Data were presented as the means ± SD. P < 0.05 was considered a significant difference.

## Results

### CIH caused injury in pancreatic tissue

**Fig 1:**
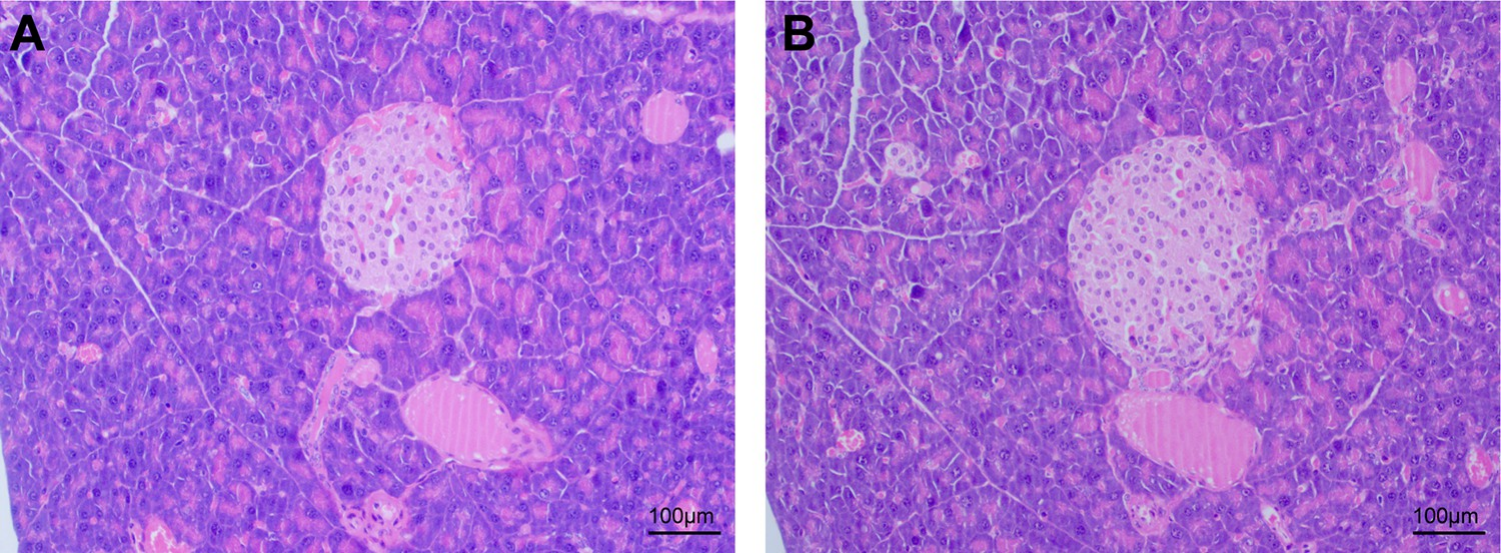
Effect of CIH on the pancreatic tissue. (A) Pancreatic histology of normoxia mice with normal architecture. (B) Pancreatic histology of CIH mice with abnormal pancreatic architecture (magnification × 100).

To observe the pathological changes in the pancreatic tissue, we performed HE staining. As shown in the Fig 1, CIH treatment resulted in obvious damage in pancreatic cells with shrunk nuclei, acinar atrophy of the pancreas, and atrophy of pancreatic islets.

### Different expression profiles of circRNAs

**Fig 2:**
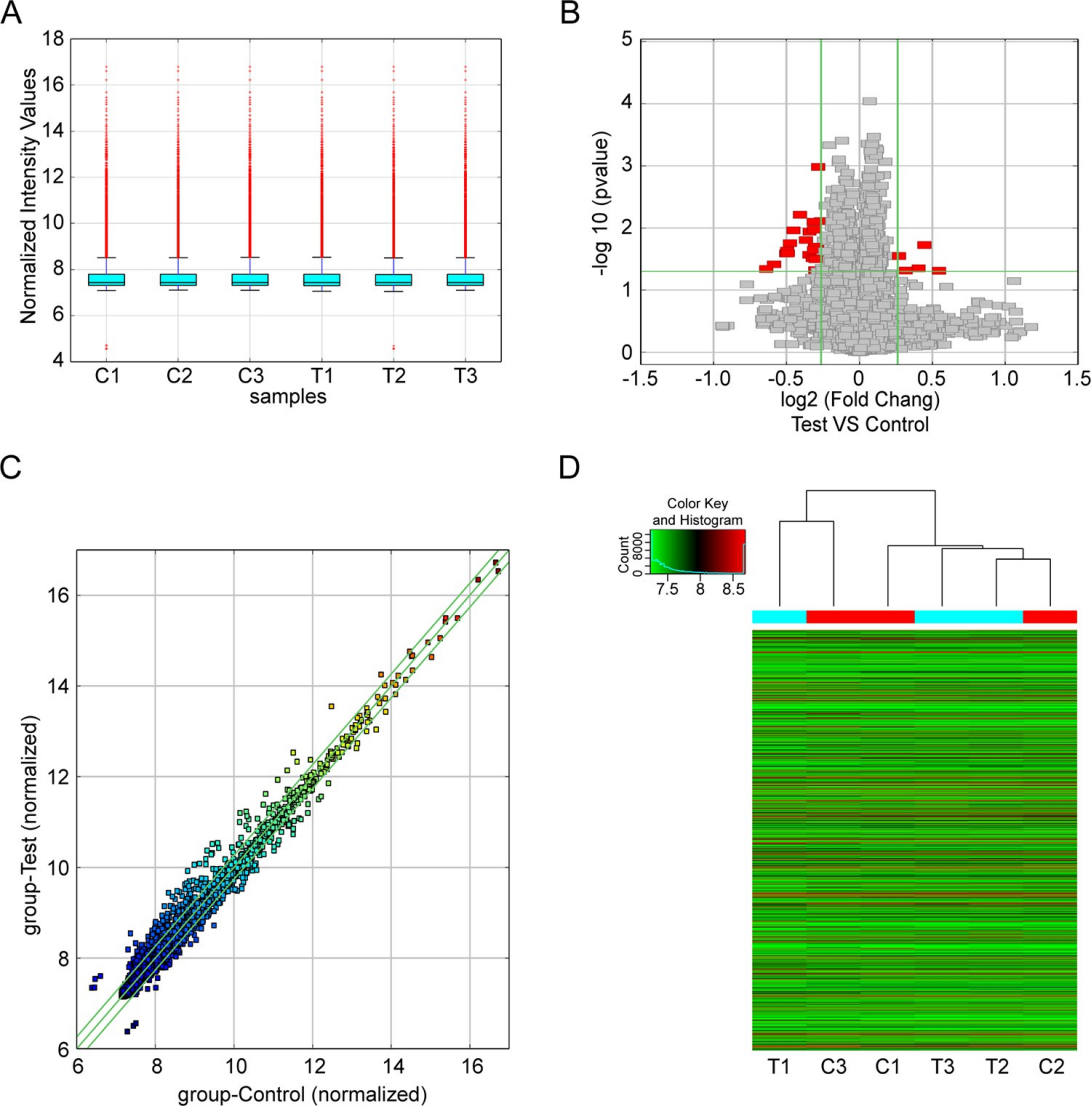
CircRNA expression patterns between CIH and control samples. (A) Box plot. circRNAs expression profiles were shown after normalization. (B) Volcano plot. The horizontal line indicates P = 0.05, while the red points indicate the altered circRNAs. (C) Scatter plot. Scatter plots indicate the difference of circRNAs expression. (D) Hierarchical cluster of all dysregulated circRNAs.

**Fig 3:**
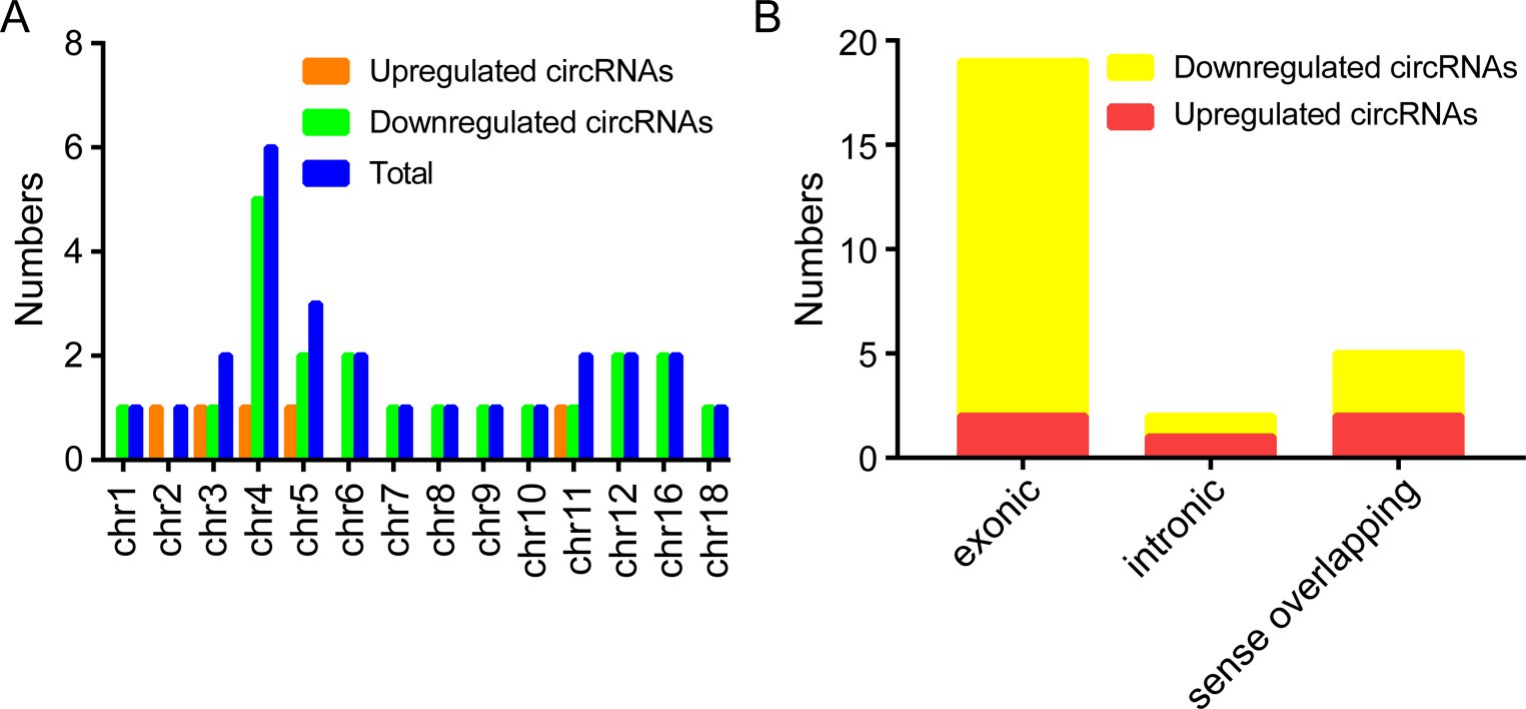
Characteristics of altered circRNAs between CIH and control samples. (A) The cluster shaped bar diagram shows the chromosome distribution of dysregulated circRNAs. (B) The histogram shows the origination of circRNAs.

A total of 26 circRNAs were found to be differentially expressed (FC ≥ 1.2 and P ≤ 0.05) in the CIH group, of which 5 were upregulated and 21 were downregulated. As shown in the box plot, the distributions of all included samples in both groups were similar (Fig 2A). The volcano and scatter plots displayed circRNA expression variation between CIH and control groups (Fig 2B and 2C). Hierarchical clustering showed the overview of circRNA expression (Fig 2D). The distribution of circRNAs in mouse chromosomes was listed in the cluster-shaped bar chart (Fig 3A). At last, we further studied the general signatures of these altered circRNAs and discovered that most of them were located in exons, while relatively few were located in introns. (Fig 3B).

### Validation of altered circRNAs

**Fig 4:**
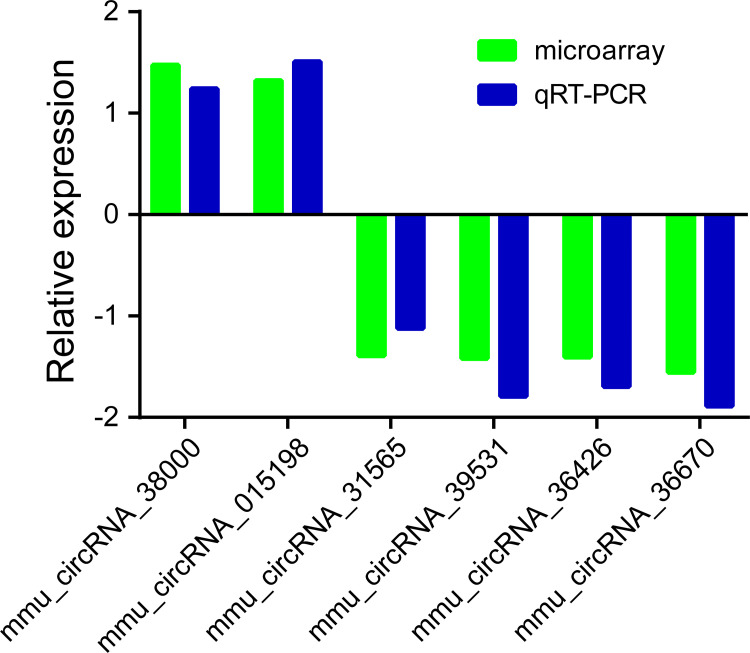
Validation results for the selected circRNAs assessed by qRT-PCR.

To confirm the circRNA microarray results, six of the significantly differentially expressed circRNAs including mmu_circRNA_38000, mmu_circRNA_015198, mmu_circRNA_31565, mmu_circRNA_36426, mmu_circRNA_39531, and mmu_circRNA_36670 were randomly selected and validated by the use of qRT-PCR (Fig 4). The qRT-PCR results demonstrated that the expressions of mmu_circRNA_38000 and mmu_circRNA_015198 were significantly increased. Likewise, mmu_circRNA_31565, mmu_circRNA_36426, mmu_circRNA_39531, and mmu_circRNA_36670 were significantly decreased. A similar trend was observed with microarray analysis, confirming the reliability of our circRNA microarray data.

### Detailed annotation for circRNA-miRNA interactions

**Fig 5:**
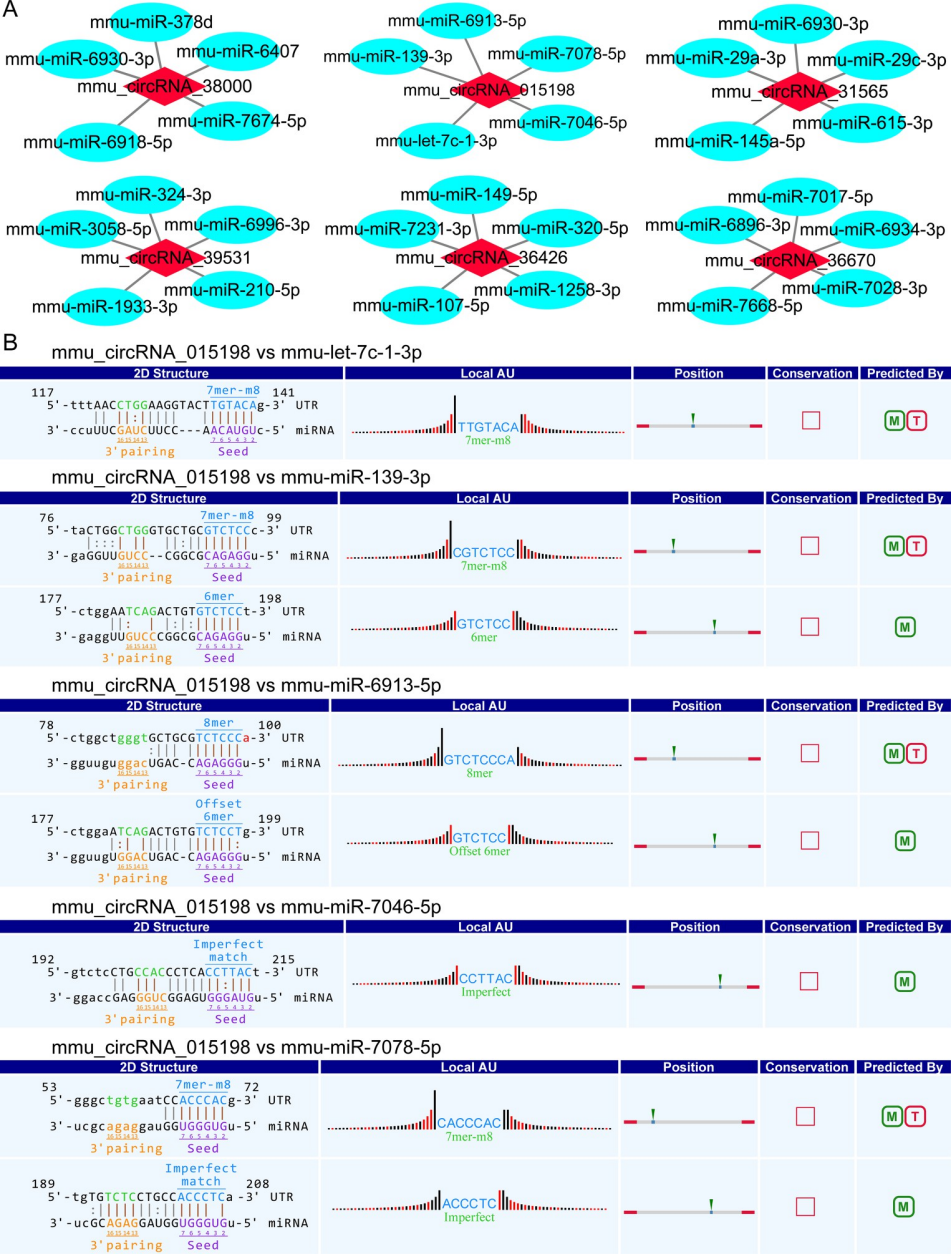
Prediction of circRNA-miRNA interaction. (A) Top 5 predicted targets of 6 validated circRNAs. (B) Predicted interaction sites of mmu_circRNA_015198. M: miRanda; T: TargetScan.

To explore the function of the validated circRNAs (mmu_circRNA_38000, mmu_circRNA_015198, mmu_circRNA_31565, mmu_circRNA_36426, mmu_circRNA_39531, and mmu_circRNA_36670), their target miRNAs were bioinformatically predicted based on miRanda and TargetScan. The top 5 miRNAs were listed in Fig 5A. Meanwhile, as shown in Fig 5B, we further displayed the predicted interaction sites of mmu_circRNA_015198.

### GO and KEGG pathway analysis

**Fig 6:**
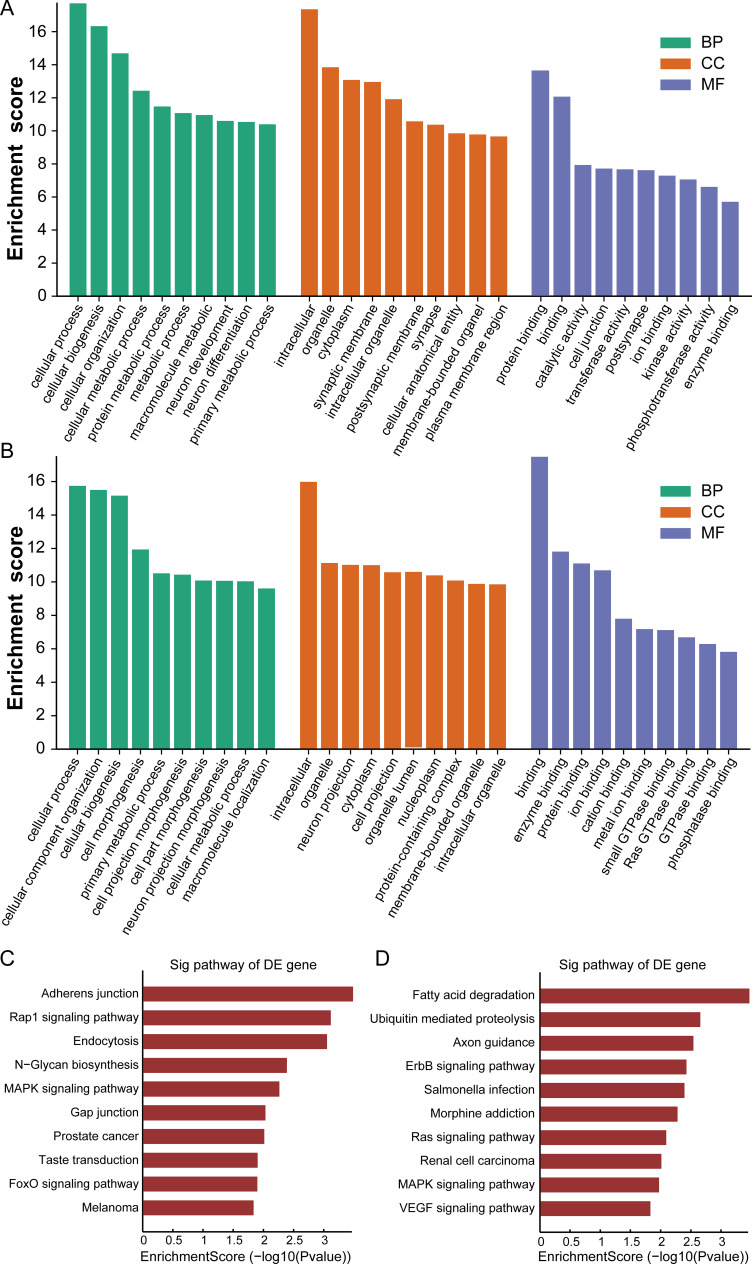
Functional analysis of validated circRNAs. (A) GO enrichment analysis of the upregulated circRNA parental genes. The Y axis represents log_10_ (P value). The X axis indicates the top 10 GO terms corresponding to the upregulated circRNAs. (B) GO enrichment analysis of the downregulated circRNA parental genes. The Y axis represents log_10_ (P value). The X axis indicates the top 10 GO terms corresponding to the upregulated circRNAs. (C) The top 10 Pathways of upregulated circRNAs are presented. (D) The top 10 Pathways of downregulated circRNAs are presented.

Both GO and KEGG analysis were applied to annotate the potential functions of the dysregulated circRNAs and mRNAs. As shown in Fig 6, the altered circRNAs and mRNAs were enriched in the GO terms of cellular components, biological processes, and molecular functions (Fig 6A and 6B). The data further showed that these target genes were largely involved in the process of cellular component organization, cellular process, intracellular, protein binding, binding and so on. Moreover, KEGG pathway analysis revealed that the targeted genes were primarily enriched in N-Glycan biosynthesis, the MAPK signaling pathway, Adherens junction, Fatty acid degradation, Rap1 signaling pathway, RAS signaling pathway, Axon guidance, and ErbB signaling pathway (Fig 6C and 6D). Of these, most of the differentially expressed circRNAs were related to the MAPK signaling pathway.

### Construction a regulatory network of ceRNA

**Fig 7:**
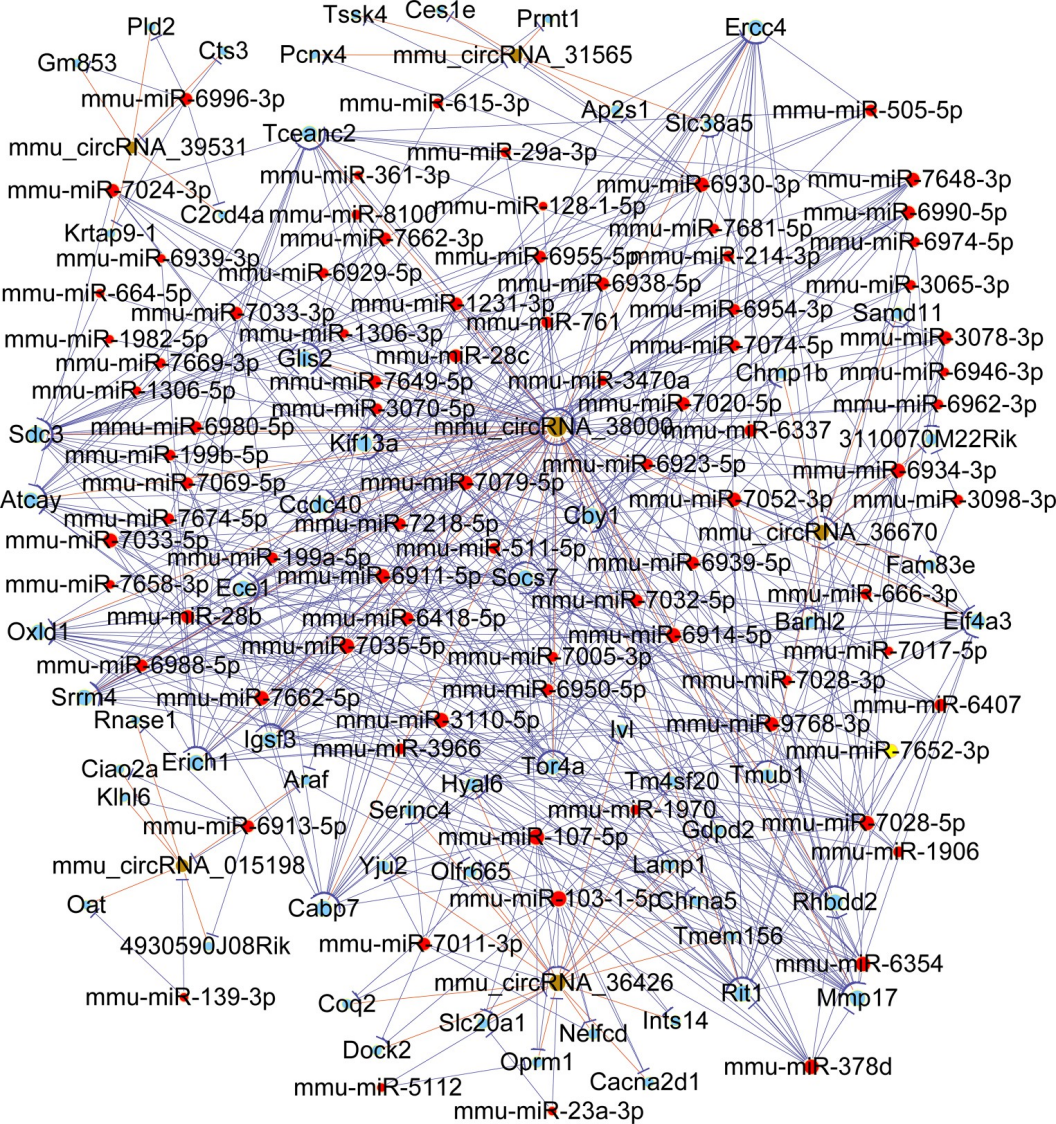
The view of circRNA-miRNA-mRNA triple network. The regulatory network contains 84 miRNAs, 6 circRNAs, and 60 mRNAs. The light-blue nodes represent mRNA, the red nodes represent miRNAs, and the brown nodes represent circRNAs.

To further explore the molecular mechanism of differentially expressed circRNAs, 6 circRNAs (mmu_circRNA_38000, mmu_circRNA_015198, mmu_circRNA_31565, mmu_circRNA_36426, mmu_circRNA_39531, and mmu_circRNA_36670) were selected to construct a circRNA-miRNA-mRNA regulatory network (Fig 7). In brief, the ceRNA network was established with 60 mRNAs, 84 predicted miRNAs, and 6 circRNAs. The network demonstrated that altered circRNAs could indirectly regulate specific miRNA target genes by acting as a sponge of miRNA via the binding sites.

## Discussion

In this study, we first systematically analyze the expression profile of circRNAs in pancreatic injury in a mouse model of CIH. The microarray data demonstrated that 21 circRNAs were significantly down-regulated, while 5 circRNAs were obviously up-regulated (FC ≥ 1.2 and P≤ 0.05). The above findings have been verified by qRT-PCR on 6 randomly selected circRNAs, including mmu_circRNA_38000, mmu_circRNA_015198, mmu_circRNA_31565, mmu_circRNA_36426, mmu_circRNA_39531, and mmu_circRNA_36670. Further bioinformatics analyses, including the GO and KEGG pathway analysis and ceRNA interaction network, were applied to predict the functions of dysregulated circRNAs, which suggested a potentially critical role of circRNAs in the pathogenesis of OSA-induced pancreatic damage.

OSA, a common sleep disorder, has a recognized prevalence of 3–7% worldwide. Accumulating evidence indicates that OSA is closely related to glucose intolerance [23], T2DM [24], insulin resistance [25], and dyslipidemia [26]. Clinical and epidemiological studies have reported that OSA can impair insulin sensitivity and/or glucose tolerance, even after adjusting for BMI. Moreover, it is suggested that CIH has a fundamental role in various metabolic impairments, including impairments in insulin sensitivity, higher fasting insulin and glucose levels, reduced beta cell function, and glucose intolerance [27–29]. In addition, it is found that the severity of metabolic dysfunction is strongly linked to the degree of sleep-related hypoxemia. To date, the underlying mechanisms of the OSA-induced glucose metabolism impairments remain to be fully elucidated.

Insight into the effects of CIH on pancreatic injury is very important for understanding the mechanisms resulting in impaired glucose homeostasis. Previous studies have found that CIH may lead to impaired pancreatic β-cell function, which provides evidence for OSA patients with a high risk of diabetes [30]. Many hypotheses have been proposed. For instance, the inflammatory response has been demonstrated to participate in CIH-induced pancreatic injury, and the levels of pro-inflammatory cytokines, IL-6, TNF-α, and IL-1β, have been shown to be enhanced by CIH [31]. Meanwhile, apoptosis has been confirmed to be involved in the underlying mechanisms of CIH-induced pancreatic β-cell death [32]. Last but not least, animal studies suggested that ROS participates in CIH-induced pancreatic damage [33]. However, the precise underlying molecular mechanism of CIH-induced pancreatic injury remains unknown.

With the development of biotechnology, a growing body of evidence revealed that circRNAs, a novel type of RNA molecules, are a stable, abundant, and conserved class of non‑coding RNAs. What’s more, recent studies showed that circRNAs could regulate a variety of human diseases, including acute ischemic stroke [34], atherosclerosis [35], cardiac fibrosis [36], and bladder carcinoma [37]. In the present study, we, for the first time, explored the circRNAs expression profiles in CIH-induced pancreatic injury in mice by using the circRNAs microarray. Our findings indicated that the expression profiles of circRNAs between the CIH group and control group were significantly different, with 21 circRNAs down-regulated and 5 circRNAs up-regulated. At the same time, we also identified the circRNAs type, and chromosome location of these differentially expressed circRNAs. In addition, 6 dysregulated circRNAs were randomly selected to confirm the microarray data by qRT-PCR. The consistency between qRT-PCR results and microarray data further supported that circRNAs played key roles in the development and progression of OSA-induced pancreatic injury.

CircRNAs can function as miRNA sponges to regulate downstream mRNA through the ceRNA network. On the basis of the ceRNA hypothesis, circRNAs can affect the activity of miRNA via sequestration, thereby decreasing or increasing the expression of miRNA target genes [38, 39]. In our study, we also built up the ceRNA crosstalk network to reveal the role of circRNAs in the mouse model of CIH-induced pancreatic injury. The six validated upregulated circRNAs (mmu_circRNA_38000, mmu_circRNA_015198, mmu_circRNA_31565, mmu_circRNA_36426, mmu_circRNA_39531, and mmu_circRNA_36670) were found to target 84 miRNAs. In our study, mmu_circRNA_015198 was obviously up-regulated, which indicated that mmu_circRNA_015198 might take part in the process of CIH-induced pancreatic injury by suppressing miRNA activity. Our circRNA/miRNA analysis further suggested that the most likely target miRNA for mmu_circRNA_015198 included mmu-let-7c-1-3p, mmu-miR-139-3p, mmu-miR-6913-5p, mmu-miR-7046-5p, and mmu-miR-7078-5p. In addition, the ceRNA network found a novel association between the dysregulated circRNAs and 60 mRNAs. Above all, this network provided credible evidence that circRNAs played an important role in the pathogenesis of OSA-induced pancreatic damage by indirectly targeting certain mRNAs.

To further explore the function of these target genes included in the ceRNA network, the GO and KEGG analysis were also performed by us. The cellular component analysis indicated that the target genes of dysregulated circRNAs were mainly associated with intracellular, organelle, and cytoplasm. The biological process analysis revealed their target genes were mainly related to cellular component organization or biogenesis, cellular component organization, and cellular process. The molecular function analysis showed that they mainly focused on protein binding, and enzyme binding. Through KEGG analysis, we found that several important pathways were linked to Adherens junction, RAS signaling pathway, and MAPK signaling pathway. Several studies reported that MAPK signaling pathways play crucial roles in inflammatory response [40, 41]. Suppressing the abnormal activation of MAPK pathways has been reported to attenuate CIH-induced inflammation [42]. Moreover, MAPK activation has also participated in CIH-induced apoptosis [7]. From these results, we suggested that MAPK pathways were involved in the regulatory mechanisms of CIH-induced pancreatic injury. Altogether, both GO and KEGG analysis also demonstrated that dysregulated circRNAs might involve in the process of CIH-related pancreatic damage via different pathways. Future investigations are needed to better elucidate the roles of these signaling pathways in OSA-induced pancreatic injury.

Although we had systemically profiled circRNA expression in the mouse model of CIH-induced pancreatic injury for the first time, our study also had several potential limitations. First and foremost, the circRNA expression profile must be interpreted cautiously due to the very small number of samples. More samples should be required in future research. Secondly, the underlying molecular regulation of circRNAs in OSA-induced pancreatic injury and their downstream mechanisms require further studies. Thirdly, the diagnostic value of circRNA levels needs to be further evaluated in other samples, such as blood samples. This will broaden the use of our findings since they might function as a diagnostic biomarker in the progression of OSA-related pancreatic injury. Fourthly, the microarray-based assays may miss out some important circRNAs, because it lacks the sensitivity of some advanced approaches such as next-generation RNA-seq techniques. Fifthly, the article does not provide evidence of insulin resistance and glucose intolerance, which will reduce the persuasiveness of our study. Last, this study was only conducted in animal models. Further investigation should be performed at the tissue, cell, and individual levels.

## Conclusions

In summary, our study first provides a preliminary landscape of the differential expression of circRNAs, which may take part in the occurrence and pathogenesis of CIH-induced pancreatic damage. Therefore, these findings may provide novel treatment strategies for OSA-induced pancreatic injury. However, we need to carry out further studies to explore the function and underlying mechanism of circRNAs in pancreatic injury induced by OSA in the future.
